# PfRH5 vaccine; from the bench to the vial

**DOI:** 10.1038/s41541-025-01137-6

**Published:** 2025-04-24

**Authors:** Philip Ilani, Prince B. Nyarko, Abdouramane Camara, Lucas N. Amenga-Etego, Yaw Aniweh

**Affiliations:** 1 West African Centre for Cell Biology of Infectious Pathogens (WACCBIP), College of Basic and Applied Sciences, University of Ghana, Legon, Accra, Ghana; 2 Laboratory of Pathogens and Host Immunity (LPHI), CNRS, University of Montpellier, Montpellier, France; 3 Present Address: ISGlobal, Hospital Clinic - Universitat de Barcelona, Barcelona, Spain

**Keywords:** Infectious diseases, Protein vaccines

## Abstract

The search for potent malaria vaccine candidate has seen several twists and turns. Here, we provide a perspective on the current state of PfRH5-based malaria vaccine development, the progress, existing challenges, and future research directions. We discuss the clinical trials in endemic regions, immune correlates of protection, prospects of integrating PfRH5 into multi-antigen vaccine strategies and considerations on the onward development/deployment of PfRH5 vaccine from the laboratory to endemic communities.

## Introduction

Malaria remains one of the world’s deadliest infectious diseases, disproportionately affecting low-income regions, particularly in sub-Saharan Africa. The disease, caused by *Plasmodium* and transmitted by the female anopheles mosquito, accounts for over 200 million cases and nearly half a million deaths annually, with pregnant women and children under five being the most vulnerable^1^. Several control measures including insecticides and insecticide-treated bed nets, frontline-line antimalarial drugs, and the licensed RTS,S/AS01 and R21/Matrix-M vaccines are valuable tools aiding disease burden reduction. The efficacy of RTS,S/AS01 is modest, with clinical trials showing approximately 36% (95% CI 31.8–40.5) and 28% (95% CI 23.3–32.9), with or without booster, respectively, in children aged 5–17 months. In young infants (age 6–12 weeks) the vaccine offered protection of about 18% without booster (95% CI 11.7–24.4) and 26% with booster (95% CI 19.9–31.5) between 2009/2011 and 2014^2^. This report indicates that efficacy wanes over time and thus require booster doses to maintain effectiveness.

Recently, the R21/Matrix-M malaria vaccine which incorporates higher CSP antigen content^3^, has also shown high protective efficacy of 75% (95% CI 71–79) in four African countries in a phase 3 trial where children aged 5–36 months were sampled from 14 days after third vaccination to 12 months post-vaccination between 2021 and 2022^4^. However, though both RTS,S/AS01 and R21/Matrix-M target the pre-erythrocytic stage, intended to prevent debilitating blood stage infection, neither provides either complete or lifelong immunity, thus limiting their impact on clinical disease severity and transmission once infection progresses to blood stage. This has spurred the search for next-generation vaccines with greater efficacy and broader protection. Among the most promising blood-stage malaria vaccine candidates is *Plasmodium falciparum* reticulocyte-binding protein homolog 5 (PfRH5) that plays a critical role during erythrocytes invasion^5,6^.

Herein, we analyse the progress in the development of PfRH5-based blood stage malaria vaccine. We discuss the variability in immune responses, antigenic diversity, and the complexities of inducing durable immunity as key issues hindering malaria vaccine effectivity^7,8^. Additionally, we touch on optimization of vaccine candidates for broader population coverage and comprehensive understanding of immunological landscape as factors that could enhance the development and deployment of PfRH5 vaccine within the shortest timeline.

## Unique aspects of PfRH5 that make it a viable vaccine candidate

**Fig. 1:**
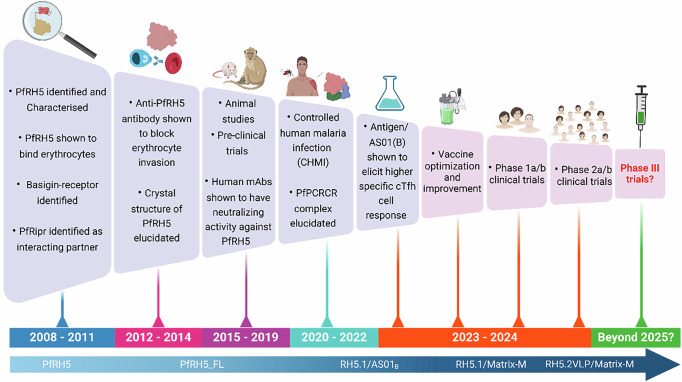
Schematic of PfRH5-based blood stage malaria vaccine development pipeline and key milestones on the journey from the lab to the bedside. The figure illustrates key findings from antigen discovery to clinical trials of the PfRH5-based vaccines. The RH5.1/Matrix-M is currently being tested in a phase 2b trial for its efficacy in children in Burkina Faso with early data showing promising outcomes. The improved RH5.2-VLP/Matrix-M vaccine is currently undergoing phase 1a/b clinical trials in the UK and The Gambia. PfRH5_FL, PfRH5 full-length antigen, CHMI Controlled Human Malaria Infection.

PfRH5 is a conserved protein that mediates a critical interaction of merozoite with the human receptor basigin (CD147), a key step in erythrocyte invasion. Unlike other proteins involved in *P. falciparum*’s erythrocyte invasion, PfRH5 is essential, with no known orthologues in *P. vivax* or other human-infecting *Plasmodium* species^9^ and exhibits minimal sequence variation in many regions^8,10,11^. Additionally, the low antigenic diversity of PfRH5 circumvents a common challenge in malaria vaccine development; the high variability and potential vulnerability for immune evasion mechanisms of many blood-stage antigens. This positions PfRH5 as a strong candidate for a broadly effective vaccine, potentially offering cross-strain protection; an advantage over other blood-stage antigens that show higher variability. These attributes represented early milestones that have translated to the most advanced blood-stage malaria vaccine (Fig. 1).

**Fig. 2:**
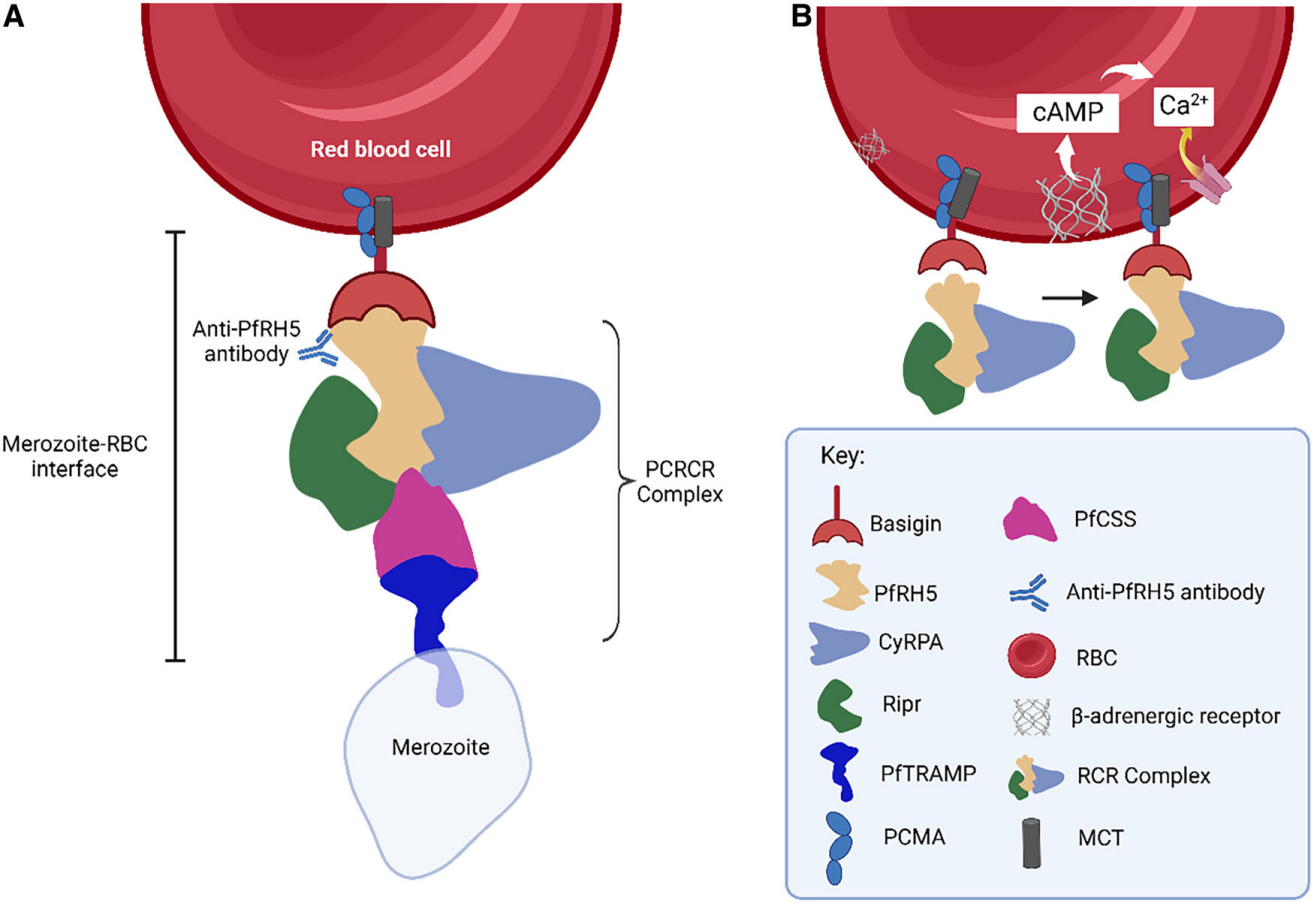
PfRH5, a leading blood stage vaccine candidate, is an essential member of the invasion complex. **A** The heteromeric complex involved in the invasion of red blood cells by *P. falciparum* merozoites. The *Plasmodium* thrombospondin-related apical merozoite protein (PTRAMP) and cysteine-rich small secreted protein (CSS) anchors the trimeric RH5-CyRPA-Ripr complex on merozoite surface. **B** PfRH5 is a nexus for signalling events. Binding of PfRH5 to its receptor basigin trigger increase in RBC cyclic adenosine monophosphate (cAMP) to promote Ca^2+^ influx, a process shown to impact junction formation and invasion of erythrocytes.

PfRH5 forms part of a complex made up of the cysteine-rich protective antigen (CyRPA) and RH5-interacting protein (Ripr), commonly called RCR (RH5-CyRPA-Ripr) complex^12,13^. The RCR complex in turn is thought to be anchored by *Plasmodium* thrombospondin-related apical merozoite protein (PTRAMP) and cysteine-rich small secreted protein (CSS) to form the pentameric complex called PTRAMP/CSS/Ripr/CyRPA/RH5 (PCRCR) at the interface between red blood cells and the infective merozoite during invasion (Fig. 2A). These complexes play a crucial role in the growth and development of the parasite^14–16^ and thus affirms it as important target for vaccine development.

## PfRH5 as a nexus for signalling events

The PCRCR complex is suggested to be required for rhoptry discharge and calcium signalling during invasion^16,17^. PfRH5-basigin interaction induces RBC Ca^2+^ influx from the extracellular milieu thus leading to changes in RBC cytoskeleton^18^. Binding of PfRH5 to its receptor basigin has recently been shown to trigger increase in RBC cyclic adenosine monophosphate (cAMP) to promote Ca^2+^ influx (Fig. 2B), thereby significantly impacting tight junction formation and invasion processes^19^. These studies provide evidence of host cell signalling events necessary for erythrocyte invasion, a conserved process across *Plasmodium* parasites. PfRH5’s likely central role in this critical process reinforces its essentiality and candidacy for an effective malaria vaccine.

## PfRH5 vaccine development and optimisation

PfRH5 is extensively being explored for the development of a blood-stage vaccine capable of providing protective immunity against the deadliest form of malaria. One notable milestone is the observation that in vitro growth inhibition activity (GIA) of purified human anti-PfRH5 IgGs correlated positively to inhibition of the in vivo parasite multiplication rate during a primary and secondary Controlled Human Malaria Infection (CHMI) post RH5.1/AS01_B_ vaccine administration^20^. Similar studies have also shown evidence of protection in mice and Aotus monkeys, thus elucidating the protective capabilities of merozoite-neutralizing PfRH5 antibodies against *P. falciparum* blood-stage infections^21,22^. These studies provided substantial evidence of protection which correlates with PfRH5 antibody levels, thus reaffirming PfRH5 as a viable vaccine target.

Over the past decade, multiple studies have identified other key features of PfRH5 that can be exploited for vaccine development, including the presence of neutralizing epitopes and strain transcending antibody activity against both laboratory and clinical parasite isolates^23–26^. Additionally, development of recombinant proteins, virus-like particles, and other delivery systems have shown potential in preclinical models and early-phase clinical trials^27–30^. These key milestones underscore the enormous research efforts geared towards developing a potent blood-stage antigen, harnessing knowledge from basic cell, molecular, and structural biology, to immunology and onward translation to clinical trials (Fig. 1).

The disruption of PfRH5-basigin interaction (Fig. 2A) by epitope-specific antibodies that can significantly inhibit erythrocyte invasion^31,32^ further highlights PfRH5’s potential as a target for an efficacious vaccine. Recent studies are beginning to reveal possible mode(s) of action(s) of PfRH5 antibodies. The physiological forms of human erythrocytic basigin is predominantly expressed in complex with monocarboxylate transporters (MCTs) or plasma membrane calcium ATPases (PMCAs)^33,34^. These complexes have been shown to exhibit greater binding affinity to PfRH5 than monomeric basigin. Surprisingly, the most effective growth-inhibitory anti-PfRH5 antibodies which do not reduce binding of full-length monomeric PfRH5 to basigin were found to inhibit binding of PfRH5 to basigin-PMCA and basigin-MCT1 complexes^31^. Thus, the likely mechanism employed by these neutralizing antibodies might involve sterically hindering the essential interaction of PfRH5 with basigin in its physiological form.

Additionally, it is becoming increasingly evident that combining PfRH5 with other invasion-related proteins, such as CyRPA and Ripr, might enhance the robustness of immune response and minimize potential immune escape mechanisms by working additively or synergistically to improve antibody activity^24,35^. This combinatorial strategy may provide a more comprehensive immune response, enhancing functional immunogenicity of a PfRH5-based vaccine as evident in preclinical settings^35^. In this respect, fusion of the C-terminal EGF-like domains of Ripr and CyRPA to form a construct termed R78C, combined with PfRH5, produced antibodies with higher GIA in vitro compared to PfRH5 alone^35^. Together, these recent results suggest that a promising strategy for an effective malaria vaccine may involve multi-antigen and/or multi-stage (e.g. PfRH5/CSP-based) vaccine combination that targets both pre-erythrocytic and blood-stages to prevent parasite infection and subsequent development in human host, respectively.

## The superiority of PfRH5 as a blood-stage vaccine target; a cautionary tale

The poor efficacy of apical membrane antigen (AMA1)-based FMP2.1/AS02A and merozoite surface protein 3 (MSP3)-based GMZ2; the blood-stage malaria vaccines that advanced to phase 2b trials^36–38^, necessitated the urgent need for a better alternative. This failure is believed to be largely due to the highly polymorphic nature of the antigens, with efforts at improving their efficacy proving abortive even with new adjuvants^39^. PfRH5 exhibit distinct immunogenic profile when compared to these other blood-stage antigens. Unlike MSP and AMA1, PfRH5’s limited polymorphism and essential role in invasion makes it less susceptible to rapid evolution of immune escape variants; a common issue in malaria vaccine development.

It is worthy of note that polymorphism in PfRH5 mediate host selection/tropism^9^. Thus, even minute genetic variation may be consequential to PfRH5 function and plausible vaccine efficacy. However, the relationship between genetic and phenotypic characteristics of naturally occurring allelic variants of PfRH5 and the impact on erythrocyte invasion remain unclear. Early data using ex vivo invasion inhibition assay coupled with next-generation sequencing revealed no statistically significant difference in invasion inhibition for any single nucleotide polymorphisms in a dose-dependent manner^8^. Although a study in Kenya identified 58 variants in the RCR protein complex with PfRH5 harbouring 30 SNPs^11^, while some SNPs are present at the PfRH5-basigin binding interface^7^, the impact of these polymorphisms on vaccine-induced polyclonal PfRH5-specific antibodies are yet to be fully understood. Whether these naturally occurring polymorphism may aid vaccine evasion, or the introduction of vaccines could induce selective pressure to expand the SNP repertoire, leading to changes in the binding characteristics of PfRH5, and if so, how long this occurrence might take remains to be addressed. While some human anti-PfRH5 monoclonal antibodies exhibit differential strain neutralisation activity^23,40^, available data still overwhelmingly support the strain-transcending activity of PfRH5 vaccine-induced (monoclonal and polyclonal) antibodies against erythrocyte invasion, as shown in genetically diverse *P. falciparum* isolates^23,25^. Thus, despite the occurrence of SNPs in the *pfrh5* gene, and the loss of inhibitory activity of some monoclonal antibodies against isolates harbouring certain PfRH5 variants, the consequence of these polymorphisms on the efficacy of vaccine-induced polyclonal antibodies remains uncertain. Nonetheless, with monoclonal antibody combinations providing potentiating effects^23,40^, suffice it to postulate that vaccine-induced polyclonal antibodies may overcome the challenge posed by PfRH5 genetic variants. This however remains to be empirically tested (at scale). Put together, PfRH5 so far shows great potential as the leading blood-stage malaria vaccine candidate when compared to other blood-stage counterparts.

## Evidence from preclinical and clinical development of PfRH5-based vaccines

### Preclinical studies

Recent studies have demonstrated that PfRH5 can generate potent immune response, leading to significant protection against *P. falciparum* infection in non-human primates and rodent models. PfRH5-specific antibodies blocked erythrocyte invasion, corroborating its viability for vaccine development^40,41^. In a study using Aotus monkeys, high titres of PfRH5 antibodies were associated with reduced parasitaemia levels^42^. These preclinical findings underscore the potential of PfRH5 as a target in vaccine design and supported its transition into clinical trials.

### Phase 1 and 2 clinical trials

The initial clinical trials of PfRH5-based vaccines focused on assessing safety, immunogenicity, and efficacy in human subjects. First human studies used a replication-deficient chimpanzee adenovirus serotype 63 (ChAd63) with an additional boost containing a modified vaccinia virus Ankara (MVA). The immunogenicity results showed that vaccinated individuals developed functional antibodies capable of inhibiting erythrocyte invasion by multiple *Plasmodium* strains in vitro^25^. In a study using viral-vectored vaccine, the immune response was observed to be higher in children compared to adults and revealed the highest functional in vitro GIA levels recorded so far after vaccination in humans^43^. The data was timely and interesting, considering that children represent the largest population affected by malaria and thus the main target group for malaria vaccine.

In a phase 1/2a clinical trial, AS01 adjuvant was used to formulate the RH5.1 (soluble full-length PfRH5 recombinant protein) vaccine. Despite offering partial protection, the vaccine showed reductions in parasite multiplication rates as observed in vaccinated participants compared to controls^20^. Subsequently, children vaccinated with RH5.1/Matrix-M vaccine (RH5.1 antigen coupled with Matrix-M adjuvant) in Tanzania produced growth inhibitory antibody levels comparable to limits previously associated with protection in non-human primates^44^. Consequently, RH5.1/Matrix-M vaccine is currently being tested in a phase 2b trial (ClinicalTrials.gov: NCT05790889) for its efficacy in children (aged 5–17 months) in Burkina Faso. Early results between April to September 2023 show that purified IgGs from the vaccinees have high in vitro GIA against *P. falciparum*. The vaccine efficacy reported in the study was estimated to be 40% (95% CI −3 to 65%) to 55% (95% CI 20 to 75%) for monthly and delayed third-dose regimen, respectively^45^.

Overall, these trials demonstrate that PfRH5 vaccines are generally safe and well-tolerated, with adverse events being mild and transient, thus supporting the viability of PfRH5 as a target for malaria vaccines and offering fundamental data for further clinical development.

### Considerations to improve PfRH5-based vaccine efficacy

In recent years, several studies have devised strategies to build upon the efficacy of RH5.1 vaccine antigen. Vaccine platforms greatly impact performance of candidate antigens in eliciting durable protective immune response. Notably, comparison of heterologous viral vector prime boost and protein coupled with AS01(B) adjuvant platforms in vaccinees shows that AS01(B) performed better at inducing a higher-magnitude humoral immune response via PfRH5-specific circulating T follicular helper (cTfh) cell response^46^. This re-emphasised the role of vaccine platforms in inducing humoral immune response.

Campeotto and colleagues computationally designed PfRH5 vaccine variants exploiting properties such as improved packing surface polarity to produce a thermally stable and immunogenic antigen^47^. This biophysical data coupled with identification of the PCRCR core that interacts with human receptor-basigin^14^ further provides critical steps in optimizing PfRH5-based vaccines and advancing its potential as an effective malaria control tool.

Additionally, a study recently characterised robust immunological landscape of PfRH5-based vaccine responders and showed that the inhibitory monoclonal antibodies induced in RH5.1/AS01B vaccinees localised around basigin-PfRH5 interface on the top half of PfRH5 structure^48,49^. This emphasizes the structural considerations required in designing the next-generation PfRH5-based vaccines and supports the idea of targeting the top half of correctly folded PfRH5 antigen where inhibitory epitopes have been localised. Potentially, this could lead to the development of a PfRH5-based vaccine with superior immune response.

One key hindrance to an efficacious malaria vaccine is the ability of candidate antigens to elicit robust and durable immune response that are effective against a wide range of field isolates and can span across different geographical regions. In asymptomatic *P. falciparum* patients from highly endemic areas of Tanzania, natural selection indices including genetic polymorphism and antigenicity screening revealed potential for positive selection and a recent population expansion of PfRH5^50^. Whether these genetic variations and natural selection pressure on PfRH5 cuts across different sub-Saharan African regions and thus potentially impact long-term effectiveness of PfRH5 vaccine on a global scale are issues that require a more robust study to understand. Thus, next generation PfRH5-based malaria vaccine development efforts as a matter of necessity should consider possibilities of the potential impact of increasing genetic variation and geographical differences on the humoral immune responses induced in vaccinees.

To ascertain whether immunological landscape in natural infection would closely mimic vaccination, a study in Mali found that anti-PfRH5 antibody levels correspond with prolonged infection albeit with rare but potently neutralizing antibodies that target same epitopes as vaccine-induced antibodies^51^. Even though antibodies from these vaccinated individuals were found to be short-lived, PfRH5 blood stage vaccine development has seen promising prospects. However, several questions remain unanswered. For instance, how natural infection contribute to PfRH5 vaccine efficacy remains a puzzle. Can natural infection serve as a booster for an efficacious PfRH5 vaccine, or would natural infection negatively impact vaccine efficacy, especially in high malaria transmission areas? Could factors such as affinity maturation of vaccine induced B cells and/or incident infections during early days post vaccination affect quality of immune responses to PfRH5 vaccines? This phenomenon was recently observed for the *Plasmodium vivax* Duffy-binding protein Matrix-M vaccine (PvDBPII/Matrix-M) where delayed booster improved antibody response compared to early boosting^52^. Improved efficacy of the RH5.1/Matrix-M upon a delayed third dose in Burkina Faso^45^, provides a case in support of delayed boosting. However, whether natural infections could serve as “early boosters” to disrupt PfRH5 vaccine efficacy remains to be properly understood and may require empirical investigation. It could be hypothesised that natural infection post-vaccination could present decoy antigens that shifts the focus from vaccine epitopes to “less important” antigens/epitopes. Addressing these seeming challenges will provide insights on strategies to adopt for malaria control using these vaccines, once licenced.

Recently, a more advanced PfRH5-based vaccine called RH5.2-VLP/Matrix-M has been developed. King and colleagues found that by deleting the disordered N-terminal region of PfRH5, thermostabilising and engineering the antigen to include just the alpha-helical core, introducing 18 mutations and conjugating this to hepatitis B surface antigen virus-like particles (VLPs); a strategy called “plug-and-display”, induces robust, high-quality immune response and GIA in rats when compared to RH5.1/Matrix-M vaccine^29^. This improved RH5.2-VLP/Matrix-M vaccine is currently undergoing phase 1a/b clinical trials in UK and Gambia (ClinicalTrials.gov: NCT05978037 and NCT05357560).

The advancement in RH5.2-VLP/Matrix-M vaccine re-emphasizes the concerted effort from a decade of research to improve PfRH5-based vaccine, which so far appears to be the most promising blood stage malaria vaccine target with the potential of being licenced within the shortest timeline (Fig. 1). Additionally, the discovery that minimising non-functional IgG responses could greatly improve vaccine antigen and thus help prioritize functional epitopes in a vaccine candidate holds enormous potential not just for malaria but other infectious diseases as well.

### Future directions for an effective PfRH5-based vaccine

A blood-stage malaria vaccine will be an important tool in the effort towards reducing infection burden and possible eradication of this prehistoric disease. The scientific community is gaining insight into issues that have impeded progress of malaria vaccines from the lab to clinical application. Advancing the efficacy of PfRH5-based malaria vaccines will likely involve next-generation strategies such as multi-subunit, multi-epitope or multi-antigen vaccines which is widely accepted as the most promising strategy.

Despite the enormous promise held by a PfRH5-based vaccine, its potency may be improved if co-administered with either of the approved pre-erythrocytic vaccines, RTS,S/AS01 and R21/Matrix-M. This approach will provide a double-layered protection where the pre-erythrocytic vaccine limits the number of sporozoites successfully invading hepatocytes and subsequently developing into hepatic merozoites, while the PfRH5 vaccine mops up any escaped merozoites that may emerge from the liver. Such a combination will be particularly helpful to children and other vulnerable groups in high transmission settings where either vaccine on their own could likely be overwhelmed by the high parasite densities which result in life-threatening severe malaria. Nonetheless, pertinent questions may need addressing. Would both vaccines be combined as a single shot or separated? While a combined shot may be cost effective, less invasive and enhance compliance, could either vaccine antigen mask immune response to the other, especially if one antigen is more immunogenic? If separate shots are required, would the sequence of administration and/or interval in-between be critical to achieving optimal protection; which vaccine/antigen comes first?

There is also compelling evidence suggesting that combining PfRH5 with other blood stage immunogens such as CyRPA and Ripr could mitigate risks associated with antigenic escape, breadth and durability of immune responses and thus synergistically enhance overall vaccine efficacy^35,53^. Interestingly, leveraging structural biology to design a malaria vaccine targeting conserved epitopes has shown promise, potentially achieving improvement in the quality of vaccine-induced antibodies^26^. Such combinations offer the potential to generate a more robust immune response by engaging multiple mechanisms of erythrocyte invasion inhibition, laying a path toward an effective and global malaria vaccine.

Additionally, ensuring scalability and affordability of PfRH5-based vaccines is critical for equitable distribution in malaria-endemic regions. Full-length PfRH5 has been expressed in bacteria, insect and mammalian cell lines^54,55^. However, expression systems that are cheap and highly scalable would be the preferred choice to meet the high global demand for a malaria vaccine. *E. coli* expression systems are the cheapest and widely used platforms with demonstrated feasibility for large-scale vaccine manufacture^56^. Based on recent data, PfRH5 viral-vectored vaccines have also shown superior antibody immunogenicity and GIA profiles^43^. Viral vectors produce strong immune responses but are expensive, require strict biosafety, and long production timelines; making them less suitable for large scale manufacturing, especially for a disease that is endemic to low-income countries. Similarly, VLPs are immunogenic and mimic viruses but their production also requires more complex cell-based protocols.

Future vaccine development pipelines must focus on refining manufacturing processes to maintain immunogenicity while reducing production costs, ensuring accessibility for public health integration in resource-limited settings where malaria is endemic^57^. This forward-looking focus highlights the importance of innovative immunogen designs, scalable production, and multivalent strategies to overcome current limitations in malaria vaccine development and deployment.

The rollout of the RTS,S/AS01 vaccine was met with limited vaccine supply and the contentious prioritization of pilot countries over countries with higher malaria burden. This challenge in RTS,S/AS01 distribution prompted the WHO to develop a framework for allocating vaccines, putting some countries on the wait list which pressured them to register the alternative R21/Matrix-M prior to WHO approval and prequalification^58,59^. However, reaching global reduction in disease burden and possible elimination would require a more robust approach to produce and equitably distribute malaria vaccines in regions where it is most needed. To achieve this, a cheap and scalable platform for producing functional vaccine antigen would support the campaign to rollout and a malaria vaccine that meet the global demand.

Pregnant women and children below 5 years in endemic countries should rationally be the first target for a protective vaccine. However, to move towards eradication, equal priority should be given to both high and low risk individuals as the later could serve as reservoirs if neglected in the vaccination campaign. Furthermore, both low and high transmission settings ought to be prioritised. However, whether a mixed vaccination strategy prioritizing high risk/high transmission regions before switching to low risk/low transmission regions later or a stand-alone successive/intermittent mass vaccination will be a better strategy to achieve adequate protection are issues to be addressed.

Thus far, only five countries (Ghana, Kenya, Malawi, Burkina Faso and Cameroon) out of the twenty approved by the Global Alliance for Vaccines and Immunization (GAVI) in sub-Sahara African region have integrated the RTS,S/AS01 vaccine into routine immunization^59^ majorly because of distribution and logistical challenges. Future malaria vaccine rollouts must consider vaccine availability, implementation strategies and prioritise high-risk, highly endemic regions while involving all stakeholders to avert some of the challenges recorded with currently approved vaccines.

## Summary

After a long period of anticipation, we now have a malaria vaccine candidate with desirable qualities, highly conserved across global *P. falciparum* endemic populations, with less likelihood of immune escape, thus reinforcing its potential efficacy across various geographic regions. The critical role of PfRH5 in erythrocyte invasion is indispensable, which allows for broader protection and affords a more durable immune response. Additionally, PfRH5-based vaccines have demonstrated cross-strain efficacy in preclinical studies, making them particularly promising for global malaria control strategies. PfRH5-based vaccines could complement pre-erythrocytic vaccines like RTS,S/AS01 and R21/Matrix-M in providing a second layer protection. Such an approach could offer enhanced protection, addressing both initial infection and reduction of blood-stage parasite replication, ultimately aiding in disease control and transmission reduction.

## Data Availability

No datasets were generated or analysed during the current study.

## References

[1] WHO. World Health Malaria Report 2024. Retrieved on 19th March from https://www.who.int/teams/global-malaria-programme/reports/world-malaria-report-2024 (2024).

[2] RTSS Clinical Trial Partnership Efficacy and safety of RTS,S/AS01 malaria vaccine with or without a booster dose in infants and children in Africa: final results of a phase 3, individually randomised, controlled trial. *Lancet* **386**, 31–45 (2015). 25913272 10.1016/S0140-6736(15)60721-8 PMC5626001

[3] Collins, K. A., Snaith, R., Cottingham, M. G., Gilbert, S. C. & Hill, A. V. S. Enhancing protective immunity to malaria with a highly immunogenic virus-like particle vaccine. *Sci. Rep.* **7**, 46621 (2017). 28422178 10.1038/srep46621 PMC5395940

[4] Datoo, M. S. et al. Safety and efficacy of malaria vaccine candidate R21/Matrix-M in African children: a multicentre, double-blind, randomised, phase 3 trial. *Lancet* **403**, 533–544 (2024). 38310910 10.1016/S0140-6736(23)02511-4 PMC7618965

[5] Rodriguez, M., Lustigman, S., Montero, E., Oksov, Y. & Lobo, C. A. PfRH5: a novel reticulocyte-binding family homolog of plasmodium falciparum that binds to the erythrocyte, and an investigation of its receptor. *PLoS One* **3**, e3300 (2008). 18827878 10.1371/journal.pone.0003300 PMC2553180

[6] Baum, J. et al. Reticulocyte-binding protein homologue 5 - an essential adhesin involved in invasion of human erythrocytes by Plasmodium falciparum. *Int J. Parasitol.* **39**, 371–380 (2009). 19000690 10.1016/j.ijpara.2008.10.006

[7] Ndwiga, L. et al. The Plasmodium falciparum Rh5 invasion protein complex reveals an excess of rare variant mutations. *Malar. J.* **20**, 278 (2021). 34162366 10.1186/s12936-021-03815-x PMC8220363

[8] Moore, A. J. et al. Assessing the functional impact of PfRh5 genetic diversity on ex vivo erythrocyte invasion inhibition. *Sci. Rep.* **11**, 2225 (2021). 33500482 10.1038/s41598-021-81711-9 PMC7838290

[9] Hayton et al. Erythrocyte binding protein PfRH5 polymorphisms determine species-specific pathways of Plasmodium falciparum invasion. *Cell Host Microbe* **4**, 40–51 (2008). 18621009 10.1016/j.chom.2008.06.001 PMC2677973

[10] Naung, M. T. et al. Global diversity and balancing selection of 23 leading Plasmodium falciparum candidate vaccine antigens. *PLoS Comput. Biol.* **18**, e1009801 (2022). 35108259 10.1371/journal.pcbi.1009801 PMC8843232

[11] Waweru, H. et al. Limited genetic variations of the Rh5-CyRPA-Ripr invasion complex in Plasmodium falciparum parasite population in selected malaria-endemic regions, Kenya. *Front. Trop. Dis.* **4**, 1102265 (2023). 38406638 10.3389/fitd.2023.1102265 PMC7615667

[12] Ragotte, R. J., Higgins, M. K. & Draper, S. J. The RH5-CyRPA-Ripr Complex as a Malaria Vaccine Target. *Trends Parasitol.* **36**, 545–559 (2020). 32359873 10.1016/j.pt.2020.04.003 PMC7246332

[13] Reddy, K. S. et al. Multiprotein complex between the GPI-anchored CyRPA with PfRH5 and PfRipr is crucial for Plasmodium falciparum erythrocyte invasion. *Proc. Natl Acad. Sci.* **112**, 1179–1184 (2015). 25583518 10.1073/pnas.1415466112 PMC4313826

[14] Farrell, B. et al. The PfRCR complex bridges malaria parasite and erythrocyte during invasion. *Nature* **625**, 578–584 (2024). 38123677 10.1038/s41586-023-06856-1 PMC10794152

[15] Scally, S. W. et al. PCRCR complex is essential for invasion of human erythrocytes by Plasmodium falciparum. *Nat. Microbiol.* **7**, 2039–2053 (2022). 36396942 10.1038/s41564-022-01261-2 PMC9712106

[16] Volz, J. C. et al. Essential Role of the PfRh5/PfRipr/CyRPA Complex during Plasmodium falciparum Invasion of Erythrocytes. *Cell Host Microbe* **20**, 60–71 (2016). 27374406 10.1016/j.chom.2016.06.004

[17] Weiss, G. E. et al. Revealing the sequence and resulting cellular morphology of receptor-ligand interactions during Plasmodium falciparum invasion of erythrocytes. *PLoS Pathog.* **11**, e1004670 (2015). 25723550 10.1371/journal.ppat.1004670 PMC4344246

[18] Aniweh, Y. et al. P. falciparum RH5-Basigin interaction induces changes in the cytoskeleton of the host RBC. *Cell. Microbiol.* **19**, e12747 (2017). 10.1111/cmi.12747 28409866

[19] Yong, J. J. M. et al. Red blood cell signaling is functionally conserved in Plasmodium invasion. *iScience* **27**, 10.1016/j.isci.2024.111052 (2024). 10.1016/j.isci.2024.111052 PMC11615254 39635131

[20] Minassian, A. M. et al. Reduced blood-stage malaria growth and immune correlates in humans following RH5 vaccination. *Medicine* **2**, 701–719.e719 (2021). 10.1016/j.medj.2021.03.014 PMC8240500 34223402

[21] Foquet, L. et al. Plasmodium falciparum liver stage infection and transition to stable blood stage infection in liver-humanized and blood-humanized FRGN KO mice enables testing of blood stage inhibitory antibodies (reticulocyte-binding protein homolog 5) in vivo. *Front. Immunol.* **9**, 524 (2018). 29593746 10.3389/fimmu.2018.00524 PMC5861195

[22] Douglas, A. D. et al. A defined mechanistic correlate of protection against Plasmodium falciparum malaria in non-human primates. *Nat. Commun.* **10**, 1953 (2019). 31028254 10.1038/s41467-019-09894-4 PMC6486575

[23] Thiam, L. G. et al. Vaccine-induced human monoclonal antibodies to PfRH5 show broadly neutralizing activity against P. falciparum clinical isolates. *NPJ Vaccines* **9**, 198 (2024). 39448626 10.1038/s41541-024-00986-x PMC11502735

[24] Singh, H. et al. Antibody Combinations Targeting the Essential Antigens CyRPA, RH5, and MSP-119 Potently Neutralize Plasmodium falciparum Clinical Isolates From India and Africa. *J. Infect. Dis.* **223**, 1953–1964 (2021). 32989463 10.1093/infdis/jiaa608 PMC8176640

[25] Payne, R. O. et al. Human vaccination against RH5 induces neutralizing antimalarial antibodies that inhibit RH5 invasion complex interactions. *JCI Insight* **2**, 10.1172/jci.insight.96381 (2017). 10.1172/jci.insight.96381 PMC5752323 29093263

[26] Harrison, T. E. et al. Rational structure-guided design of a blood stage malaria vaccine immunogen presenting a single epitope from PfRH5. *EMBO Mol. Med.* **16**, 2539–2559 (2024). 39223355 10.1038/s44321-024-00123-0 PMC11473951

[27] Jin, J. et al. Production, quality control, stability, and potency of cGMP-produced Plasmodium falciparum RH5.1 protein vaccine expressed in Drosophila S2 cells. *NPJ Vaccines* **3**, 32 (2018). 30131879 10.1038/s41541-018-0071-7 PMC6098134

[28] Sun, Y. et al. Vesicular stomatitis virus-based vaccine targeting plasmodium blood-stage antigens elicits immune response and protects against malaria with protein booster strategy. *Front. Microbiol.* **13**, 1042414 (2022). 36504817 10.3389/fmicb.2022.1042414 PMC9731671

[29] King, L. D. W. et al. Preclinical development of a stabilized RH5 virus-like particle vaccine that induces improved antimalarial antibodies. *Cell Rep. Med.* **5**, 101654 (2024). 39019011 10.1016/j.xcrm.2024.101654 PMC11293324

[30] Flynn, O. et al. Low Adenovirus Vaccine Doses Administered to Skin Using Microneedle Patches Induce Better Functional Antibody Immunogenicity as Compared to Systemic Injection. *Vaccines* **9**, 10.3390/vaccines9030299 (2021). 10.3390/vaccines9030299 PMC8005075 33810085

[31] Jamwal, A. et al. Erythrocyte invasion-neutralising antibodies prevent Plasmodium falciparum RH5 from binding to basigin-containing membrane protein complexes. *Elife* **12**, 10.7554/eLife.83681 (2023). 10.7554/eLife.83681 PMC10569788 37796723

[32] Miura, K. et al. Assessment of precision in growth inhibition assay (GIA) using human anti-PfRH5 antibodies. *Malar. J.* **22**, 159 (2023). 37208733 10.1186/s12936-023-04591-6 PMC10196285

[33] Muramatsu, T. Basigin (CD147), a multifunctional transmembrane glycoprotein with various binding partners. *J. Biochem.* **159**, 481–490 (2015). 26684586 10.1093/jb/mvv127 PMC4846773

[34] Supper, V. et al. Association of CD147 and Calcium Exporter PMCA4 Uncouples IL-2 Expression from Early TCR Signaling. *J. Immunol.* **196**, 1387–1399 (2016). 26729804 10.4049/jimmunol.1501889

[35] Williams, B. G. et al. Development of an improved blood-stage malaria vaccine targeting the essential RH5-CyRPA-RIPR invasion complex. *Nat. Commun.* **15**, 4857 (2024). 38849365 10.1038/s41467-024-48721-3 PMC11161584

[36] Laurens, M. B. et al. Extended Safety, Immunogenicity and Efficacy of a Blood-Stage Malaria Vaccine in Malian Children: 24-Month Follow-Up of a Randomized, Double-Blinded Phase 2 Trial. *PLOS ONE* **8**, e79323 (2013). 24260195 10.1371/journal.pone.0079323 PMC3832522

[37] Nouatin, O. et al. Cellular and antibody response in GMZ2-vaccinated Gabonese volunteers in a controlled human malaria infection trial. *Malar. J.* **21**, 191 (2022). 35715803 10.1186/s12936-022-04169-8 PMC9204906

[38] Sirima, S. B. et al. A phase 2b randomized, controlled trial of the efficacy of the GMZ2 malaria vaccine in African children. *Vaccine* **34**, 4536–4542 (2016). 27477844 10.1016/j.vaccine.2016.07.041

[39] Alves, K. C. S., Guimarães, J. M., Almeida, M. E. M. D. & Mariúba, L. A. M. Plasmodium falciparum merozoite surface protein 3 as a vaccine candidate: A brief review. *Rev. do Inst. de. Med. Tropical de. São Paulo* **64**, e23 (2022). 10.1590/S1678-9946202264023 PMC8916589 35293561

[40] Alanine, D. G. W. et al. Human Antibodies that Slow Erythrocyte Invasion Potentiate Malaria-Neutralizing Antibodies. *Cell* **178**, 216–228.e221 (2019). 31204103 10.1016/j.cell.2019.05.025 PMC6602525

[41] Douglas, A. D. et al. The blood-stage malaria antigen PfRH5 is susceptible to vaccine-inducible cross-strain neutralizing antibody. *Nat. Commun.* **2**, 601 (2011). 22186897 10.1038/ncomms1615 PMC3504505

[42] Douglas, A. D. et al. A PfRH5-based vaccine is efficacious against heterologous strain blood-stage Plasmodium falciparum infection in aotus monkeys. *Cell Host Microbe* **17**, 130–139 (2015). 25590760 10.1016/j.chom.2014.11.017 PMC4297294

[43] Silk, S. E. et al. Superior antibody immunogenicity of a viral-vectored RH5 blood-stage malaria vaccine in Tanzanian infants as compared to adults. *Medicine* **4**, 668–686.e667 (2023). 10.1016/j.medj.2023.07.003 37572659

[44] Silk, S. E. et al. Blood-stage malaria vaccine candidate RH5.1/Matrix-M in healthy Tanzanian adults and children; an open-label, non-randomised, first-in-human, single-centre, phase 1b trial. *Lancet Infect. Dis.* **24**, 1105–1117 (2024). 38880111 10.1016/S1473-3099(24)00312-8 PMC12184842

[45] Natama, H. M. et al. Safety and efficacy of the blood-stage malaria vaccine RH5.1/Matrix-M in Burkina Faso: interim results of a double-blind, randomised, controlled, phase 2b trial in children. *Lancet Infect. Dis.*10.1016/s1473-3099(24)00752-7 (2024). 39672183 10.1016/S1473-3099(24)00752-7

[46] Nielsen, C. M. et al. Protein/AS01(B) vaccination elicits stronger, more Th2-skewed antigen-specific human T follicular helper cell responses than heterologous viral vectors. *Cell Rep. Med.* **2**, 100207 (2021). 33763653 10.1016/j.xcrm.2021.100207 PMC7974546

[47] Campeotto, I. et al. One-step design of a stable variant of the malaria invasion protein RH5 for use as a vaccine immunogen. *Proc. Natl Acad. Sci.* **114**, 998–1002 (2017). 28096331 10.1073/pnas.1616903114 PMC5293100

[48] Takashima, E. & Tsuboi, T. RH5: rationally-designed malaria vaccine antigen improving efficacy. *Trends Parasitol.* **40**, 870–872 (2024). 39277508 10.1016/j.pt.2024.09.001

[49] Barrett, J. R. et al. Analysis of the diverse antigenic landscape of the malaria protein RH5 identifies a potent vaccine-induced human public antibody clonotype. *Cell* **187**, 4964–4980.e4921 (2024). 39059380 10.1016/j.cell.2024.06.015 PMC11380582

[50] Jun, H. et al. Estimation of PfRh5-based vaccine efficacy in asymptomatic Plasmodium falciparum patients from high-endemic areas of Tanzania using genetic and antigenicity variation screening. *Front. Immunol.* **15**, 10.3389/fimmu.2024.1495513 (2024). 10.3389/fimmu.2024.1495513 PMC11609159 39624090

[51] Wang, L. T. et al. Natural malaria infection elicits rare but potent neutralizing antibodies to the blood-stage antigen RH5. *Cell* **187**, 4981–4995.e4914 (2024). 39059381 10.1016/j.cell.2024.06.037 PMC11383431

[52] Hou, M. M. et al. Vaccination with Plasmodium vivax Duffy-binding protein inhibits parasite growth during controlled human malaria infection. *Sci. Transl. Med.* **15**, eadf1782 (2023). 37437014 10.1126/scitranslmed.adf1782 PMC7615121

[53] Bustamante, L. Y. et al. Synergistic malaria vaccine combinations identified by systematic antigen screening. *Proc. Natl Acad. Sci. USA* **114**, 12045–12050 (2017). 29078270 10.1073/pnas.1702944114 PMC5692528

[54] Bustamante, L. Y. et al. A full-length recombinant Plasmodium falciparum PfRH5 protein induces inhibitory antibodies that are effective across common PfRH5 genetic variants. *Vaccine* **31**, 373–379 (2013). 23146673 10.1016/j.vaccine.2012.10.106 PMC3538003

[55] Reddy, K. S. et al. Bacterially expressed full-length recombinant Plasmodium falciparum RH5 protein binds erythrocytes and elicits potent strain-transcending parasite-neutralizing antibodies. *Infect. Immun.* **82**, 152–164 (2014). 24126527 10.1128/IAI.00970-13 PMC3911863

[56] Raghuwanshi, A. S. et al. Development of a process for large scale production of PfRH5 in E. coli expression system. *Int. J. Biol. Macromol.* **188**, 169–179 (2021). 34364940 10.1016/j.ijbiomac.2021.08.014

[57] Miura, K., Flores-Garcia, Y., Long, C. A. & Zavala, F. Vaccines and monoclonal antibodies: new tools for malaria control. *Clin. Microbiol. Rev.* **37**, e0007123 (2024). 38656211 10.1128/cmr.00071-23 PMC11237600

[58] WHO. *Framework for the allocation of limited malaria vaccine supply* (World Health Organization, 2022).

[59] Osoro, C. B. et al. Policy uptake and implementation of the RTS,S/AS01 malaria vaccine in sub-Saharan African countries: status 2 years following the WHO recommendation. *BMJ Glob. Health* **9**, 10.1136/bmjgh-2023-014719 (2024). 10.1136/bmjgh-2023-014719 PMC11085798 38688566

